# Risk Factors and Outcomes of Community-Acquired Carbapenem-Resistant *Klebsiella pneumoniae* Infection in Elderly Patients

**DOI:** 10.3390/antibiotics13030282

**Published:** 2024-03-20

**Authors:** Yen-Chou Chen, I-Ting Tsai, Chung-Hsu Lai, Kuo-Hsuan Lin, Yin-Chou Hsu

**Affiliations:** 1 Department of Emergency Medicine, E-Da Hospital, I-Shou University, Kaohsiung 82445, Taiwan; 2 School of Medicine, College of Medicine, I-Shou University, Kaohsiung 82445, Taiwan; 3 Division of Infectious Diseases, Department of Internal Medicine, E-Da Hospital, I-Shou University, Kaohsiung 82445, Taiwan; 4 School of Chinese Medicine for Post Baccalaureate, I-Shou University, Kaohsiung 82445, Taiwan; 5 School of Medicine for International Student, I-Shou University, Kaohsiung 82445, Taiwan; 6 Graduate Institute of Clinical Medicine, College of Medicine, Kaohsiung Medical University, Kaohsiung 80708, Taiwan

**Keywords:** elderly, *Klebsiella pneumoniae*, antibiotic resistance, carbapenem-resistant, risk factors, outcomes

## Abstract

The increasing prevalence of carbapenem-resistant *Klebsiella pneumoniae* (CRKP) infections is a global concern. Elderly patients have a diminished immune response and functional reserve, and are thus more vulnerable to bacterial infection. This study aimed to investigate the risk factors and outcomes in elderly patients with community-acquired CRKP infections. We performed a retrospective cohort study in a tertiary medical center between 1 January 2021, and 31 December 2021. All elderly patients who visited the emergency department during this period with culture-positive *K. pneumoniae* were enrolled, and their baseline demographics, laboratory profiles, management strategies, and outcomes were recorded and analyzed. We identified 528 elderly patients with *K. pneumonia* infection, and the proportion of patients with CRKP infection was 10.2% (54/528). Recent intensive care unit (ICU) admission and prior carbapenem use are independent risk factors for CRKP infection in elderly patients. Compared to patients with carbapenem-sensitive *K. pneumoniae* infection, those with CRKP infection had a significantly higher risk of adverse outcomes, including ICU care, respiratory failure, septic shock, and 90-day mortality. CRKP infection was also identified as an independent risk factor for 90-day mortality. Clinicians should be aware of the increasing prevalence of CRKP infections in elderly patients and judiciously choose appropriate antibiotics for these patients.

## 1. Introduction

*Klebsiella pneumoniae* is a Gram-negative, encapsulated, nonmotile bacterium that usually resides in the natural environment and the human mucosal surface as a benign colonization. *K. pneumoniae* is considered an opportunistic pathogen that causes a broad spectrum of diseases, especially in immunocompromised conditions [1,2]. Antibiotic treatment regimens remain the cornerstone of control strategies against *K. pneumoniae* [3]. Nevertheless, the rapidly growing prevalence of extended-spectrum β-lactamases and carbapenem-resistant strains have aroused globally widespread infection outbreaks [4,5]. The emergence of hypervirulent and antibiotic-resistant *K. pneumoniae* strains has imposed an urgent threat to global public health, with a prevalence estimated to be approximately 25% among *K. pneumoniae* clinical isolates, and a mortality rate of 42.1% in patients with carbapenem-resistant *K. pneumoniae* (CRKP) infection [1,4,6].

Elderly people have decreased immune function and responses, known as immunosenescence, which occurs through multiple mechanisms, including decreased cytokine production, altered toll-like receptor expression, thymic involution, and decreased B cells antibody affinity, all of which predispose elderly patients to infection with a more severe outcome [7,8]. Furthermore, elderly patients with multiple comorbidities frequently present with atypical and nonspecific symptoms while they have infection, such as lack of fever or chills, making prompt diagnosis and management initiation more challenging [7,9]. Nearly half of all sepsis diagnoses are estimated to be made in the elderly population [7,8].

*K. pneumoniae* is one of the most common causative microorganisms in elderly patients with infections, especially in patients with respiratory tract and bloodstream infections [10,11]. Moreover, higher morbidity and mortality rates were observed for infections sustained by multidrug resistant strains, due to the reduced therapeutic options [11]. Elderly patients not only have a higher risk of infectious disease, but are also easily colonized by multi-drug resistant strains, particularly those caused by *K. pneumoniae* in those with multiple comorbidities [12]. Extensive studies have focused on the risk factors for hospital-acquired CRKP infection in elderly patients in recent years, revealing that prior antibiotic administration (e.g., cephalosporin, fluoroquinolone, and carbapenems), comorbidities (e.g., diabetes mellitus, malignancy, and cardiovascular disease), and medical interventions (e.g., mechanical ventilation and central venous catheter insertion) were common risk factors [11,13]. Notably, few studies have investigated the risk factors and outcomes of community-acquired CRKP in this frail population. This study aimed to explore the risk factors for community-acquired CRKP infection in elderly patients and analyze their outcome predictors.

## 2. Results

**Figure 1:**
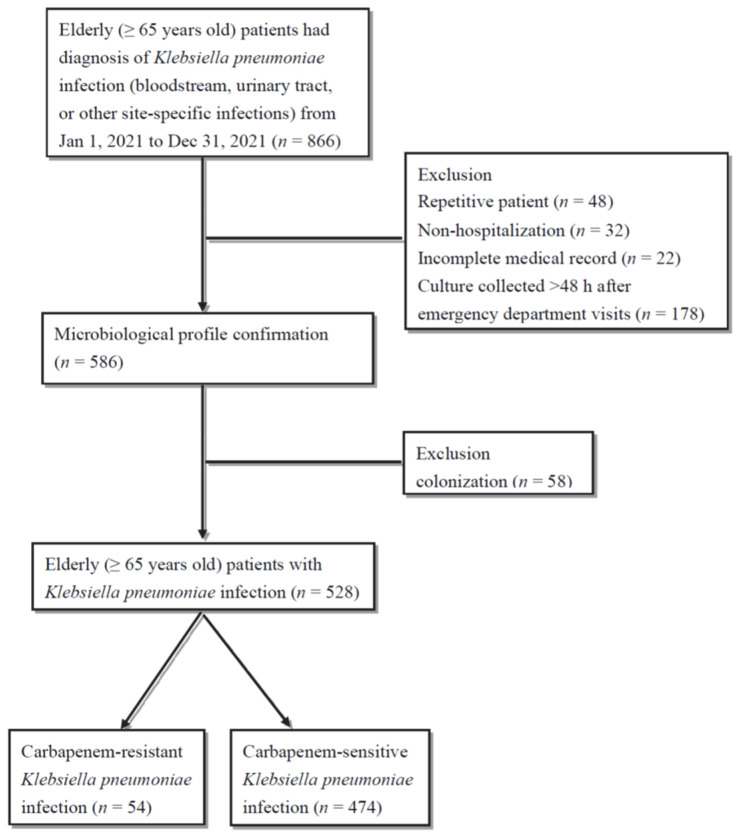
Flowchart of patient enrollment from 1 January 2021, to 31 December 2021 at E-Da Hospital, Kaohsiung, Taiwan.

Initially, 866 elderly patients with *K. pneumoniae* infections were identified during the study period (1 January 2021, to 31 December 2021). After excluding patients with multiple emergency department (ED) visits (*n* = 48), non-hospitalization (*n* = 32), incomplete medical records (*n* = 22), cultures collected >48 h after ED visits (*n* = 178), and confirmed *K. pneumoniae* colonization (*n* = 58, reviewed and confirmed by C.-H.L.), the remaining 528 patients (CRKP = 54, carbapenem-sensitive *K. pneumoniae* (CSKP) = 474) were included in the final analysis (Figure 1).

### 2.1. Clinical Characteristics of Elderly Patients with K. pneumoniae Infection

The mean age of all enrolled patients was 77.1 ± 8.2 years, and 52.5% (277/528) of them were male patients. No significant differences were present in the proportion of comorbidities or infection source distributions between the CRKP and CSKP groups (Table 1). Notably, patients in the CRKP group had a significantly higher proportion of recent intensive care unit (ICU) admissions than those in the CSKP group (50.0% vs. 13.7%, *p* < 0.01). A significantly higher proportion of prior antibiotic use was present in the CRKP group, including penicillin (24.1% vs. 13.3%, *p* = 0.04) and carbapenem (14.8% vs. 7.2%, *p* = 0.04). No significant differences were present between the two groups in the laboratory analysis, except for a significantly lower level of hemoglobin in the CRKP group (10.3 [8.8–12.4] vs. 11.5 [9.6–13.3], *p* = 0.02). The patients in the CRKP group had significantly higher sepsis-related organ failure assessment (SOFA) scores than the CSKP group (5 ± 3 vs. 4 ± 3, *p* = 0.03), indicating their disease severity (Table 1).

### 2.2. Risk Factors of CRKP Infection in Elderly Patients

Next, we investigated the risk factors for community-acquired CRKP infection in elderly patients. As shown in Table 2, we identified that recent ICU admission and prior penicillin and carbapenem use were significant factors of CRKP infection in the univariate analysis, and recent ICU admission (odds ratio (OR) = 7.48, *p* < 0.01) and prior carbapenem use (OR = 4.01, *p* = 0.02) remained independent factors of CRKP infection in the multivariate regression analysis (Table 2).

### 2.3. Outcome Analysis of Elderly Patients with K. pneumoniae Infection

Next, we analyzed the outcomes of the elderly patients with *K. pneumoniae* infection. As shown in Table 3, the overall 90-day mortality rate of the enrolled patients was 18.4% (97/528). Compared to patients in the CSKP group, those in the CRKP group had a significantly higher risk of ICU care (31.5% vs. 19.2%, *p* = 0.048), respiratory failure (29.6% vs. 14.3%, *p* < 0.01), septic shock (35.2% vs. 21.5%, *p* = 0.03), and 90-day mortality (33.3% vs. 16.6%, *p* < 0.01). Thus, the CRKP group had a significantly higher risk of all adverse outcomes than the CSKP group.

### 2.4. Risk Factors of 90-Day Mortality in Elderly Patients with K. pneumoniae Infection

Finally, we investigated the risk factors of 90-day mortality in elderly patients with *K. pneumoniae* infection. As shown in Table 4, chronic kidney disease, malignancy, CRKP strain infection, recent ICU admission, bloodstream infection, urinary tract infection, respiratory tract infection, C-reactive protein level, creatinine level, and SOFA score were significant factors for 90-day mortality in the univariate analysis. In the multivariate regression analysis, we identified malignancy (OR = 1.98, *p* < 0.01), CRKP infection (OR = 2.35, *p* = 0.04), and SOFA score (OR = 1.64, *p* < 0.01) as independent risk factors for 90-day mortality in elderly patients with *K. pneumoniae* infection (Table 4).

## 3. Discussion

In this single-center, retrospective cohort study, we described the characteristics and clinical outcomes of elderly patients with community-acquired *K. pneumoniae* infections. We also investigated the risk factors for CRKP infection and 90-day mortality in the enrolled patients. We demonstrated that, compared to patients in the CSKP group, patients in the CRKP group had significantly higher disease severity and risk of adverse outcomes, including ICU care, respiratory failure, septic shock, and 90-day mortality. We found that recent ICU admission and prior carbapenem use were risk factors for CRKP infection, whereas the comorbidities of malignancy, CRKP infection, and SOFA score were risk factors for 90-day mortality.

The emergence of CRKP is primarily from the different types of carbapenemase production (*K. pneumoniae* carbapenemases (KPC), New Delhi metallo-β-lactamase (NDM), Verona integron-encoded metallo-β-lactamase (VIM), imipenemase metallo-β-lactamase (IMP), and oxacillinase-48-type carbapenemases (OXA-48)), which further reduce the therapeutic option and increase the mortality risk. [3,12,14,15]. Furthermore, efflux pump overexpression, decreased outer membrane protein permeability, and beta-lactamase production are all important mechanisms for CRKP strain formation [1,3]. Previous studies revealed that the source of CRKP infection varies geographically in different regions [16,17]. Several Chinese studies have revealed that CRKP is a major causative microorganism of lower respiratory tract infections, whereas US-based studies have shown that CRKP is mainly derived from the urine [14,18]. Our study demonstrated that the prominence of CRKP in the bloodstream and urine was in accordance with previous meta-analyses, indicating its importance as an infection source [4,19].

Compared to younger adults, older adults have a higher risk of drug-resistant microorganism infection, which is attributed to their multiple comorbidities, higher rates of antibiotic exposure and hospitalization, and indwelling catheters [7]. Reportedly, more than half of elderly patients with infection receive ineffective antibiotic therapy because of the high prevalence of resistant pathogens [7]. Judicious use, modification, and de-escalation of antibiotics based on culture data and local antibiotic susceptibility patterns are crucial for adherence to antibiotic stewardship, thus minimizing the risk of antibiotic resistance [7,16]. Notably, past studies regarding CRKP infection in elderly patients mainly focused on hospital-acquired issues, whereas approximately 0.04–29.5% of carbapenem-resistant pathogens were reported to be of community-origin, and the trend is continually rising [20]. Our study provides evidence that patients with community-acquired CRKP infection may present similar hospital-acquired clinical characteristics and outcomes and reminds clinicians of the threat of community-acquired resistant-pathogens [10,11,13].

The rapidly increasing prevalence and spread of CRKP strains in recent years have become an immediate health threat worldwide and are estimated to be as high as 65.6% in certain regions according to a recent review [2]. Notably, two meta-analyses recognized that ICU admission, antibiotic exposure (especially carbapenems), and immunosuppression are common risk factors for CRKP infections [21,22]. The ICU environment admits the vulnerable patients who receive invasive medical interventions and antibiotic administration, and airborne and close contact can facilitate the rapid transmission of resistant pathogens [22]. Carbapenem is one of the most commonly used last-resort antibiotics for critically ill patients, further strengthening its important role in the emergence of resistant bacteria [21,22]. Similar to these findings, a recent study on elderly patients showed that comorbidities, including immunosuppressive status, ICU admission, mechanical ventilation, and prior antibiotic use were independent risk factors for CRKP infection [13]. Our study confirms that ICU admission and prior carbapenem exposure are also attributable to community-acquired CRKP infection in elderly patients, emphasizing the importance of judicious antibiotic choice in these high-risk patients.

Previous studies of CRKP infection in elderly patients have mainly focused on its association with mortality risk [10,11]. Our study highlights that the patients with CRKP infection also had a significantly higher risk of other adverse outcomes, including ICU care, respiratory failure, and septic shock, which correlated with disease severity (i.e., SOFA score). The antibiotic-resistant strain is speculated to stimulate the production of pro- and anti-inflammatory cytokines, causing an imbalance between immune activation and immunosuppression, further immunological paralysis, and finally, inducing vital organ failure (e.g., encephalopathy, acute respiratory distress syndrome, acute kidney injury, and circulatory shock) [23]. The elderly are more susceptible to these infections owing to their immunosenescence and decline in vital organ function; therefore, the CRKP group, unsurprisingly, had a higher risk of these adverse outcomes. Furthermore, the mortality risk in the CRKP group in our study is consistent with previous results [4].

Our study demonstrates that malignant comorbidities and SOFA scores were independent risk factors for the 90-day mortality of *K. pneumoniae* infection in elderly patients. The increased mortality risk in patients with malignancy can be explained by the disease itself, invasive procedures used, and immunocompromised status due to therapy [24,25]. All of these effects can inhibit the immune system, including via the pleiotropic dysregulation of innate and adaptive immune responses and decrease in white blood cells activities, further diminishing local defense mechanisms and organ function [24,25]. The SOFA score is a well-validated scoring system for prognostic stratification in patients with infection and has moderate predictive ability in ventilated patients with elderly patients with community-acquired pneumonia caused by *K. pneumoniae* [26]. Our study provides evidence that the SOFA score also plays a prognostic role in elderly patients with various types of *K. pneumoniae* infections, further broadening its potential for clinical application.

Early and appropriate antibiotic administration is the cornerstone of sepsis treatment strategies, whereas inappropriate antimicrobial therapy is an independent mortality predictor, particularly in elderly patients [8,27]. Notably, no gold standard antibiotic regimens are available for CRKP infection, and the limited treatment choices largely depend on the infection site, carbapenemase type, and isolate susceptibility results [28]. Receiving combinatory antibiotics regimen with at least two drugs with tested clinical microbiology laboratories activity has shown greater effectiveness for CRKP infection in critically ill patients [28]. Previous studies on hospital-acquired *K. pneumoniae* infections in elderly patients did not recognize CRKP infection as an independent predictor of mortality [10,11]. Our study revealed that CRKP infection was an independent risk factor for 90-day mortality in elderly patients with *K. pneumoniae* infection and is a reminder to clinicians of the importance of resistant pathogens and the judicious selection of appropriate antibiotic regimens, especially for those at risk of CRKP infection (e.g., recent ICU admission and prior carbapenem use).

This study has some limitations, including the retrospective nature of the study conducted in a single center, which limits the generalizability of these findings, and some unknown confounders were inevitably missing. The timing of antibiotic administration (i.e., the time from infection recognition to the first dose of intravenous antibiotic administration) was not calculated, and the appropriateness of empiric antibiotic therapy was not defined or analyzed in this study. We did not investigate other elements of the sepsis treatment strategy, including fluid administration and the implementation of source control measures, which can bias our results. Finally, we did not perform a molecular characterization of all clinical specimens in this study; therefore, we were not able to report the exact virulence traits, resistance mechanisms, or transferability, nor the clonal relatedness of the strains.

In conclusion, a history of recent ICU admission and carbapenem use were risk factors for CRKP infection in elderly patients with community-acquired *K. pneumoniae* infection. These patients with CRKP infection had a significantly higher risk of adverse clinical outcomes, including ICU care, respiratory failure, septic shock, and 90-day mortality. Malignant comorbidity, CRKP infection, and SOFA scores were independent predictors of mortality in these patients. Clinicians should be aware of those at risk of resistance to pathogen infection and be more active in the surveillance measures of these multidrug-resistant bacteria for prevention and control.

## 4. Materials and Methods

### 4.1. Study Design and Patient Selection

We performed a retrospective observational cohort study at E-Da Hospital, Kaohsiung, Taiwan; this is a tertiary referral medical center with 950 licensed beds and 60 adult ICU beds. Approximately 57,000 ED visits are made annually. As the definition of community-acquired infection is infection acquired outside the hospital or within 48 h of hospital admission, we enrolled all elderly patients (aged ≥ 65 years) who visited the ED from 1 January 2021, to 31 December 2021, and received blood or other site-specific cultures within 48 h of ED visit, which yielded *K. pneumoniae* in this study [29]. We included only the first visit in the study if a patient had multiple ED visits during this period. We excluded patients with incomplete medical records, non-hospitalization, cultures prepared >48 h after ED visits (i.e., hospital-acquired infections), and those deemed to be colonized by *K. pneumoniae* [30]. The specific types of infections were defined followed by the Centers for Disease Control and Prevention/National Healthcare Safety Network’s definitions (e.g., bloodstream infection: the patient has a recognized pathogen cultured from one or more blood cultures, and the organism cultured from blood is not related to an infection at another site); otherwise, they were deemed as colonization [31,32]. This study was approved by the Institutional Review Board of E-Da Hospital (EMRP-112-146) and conducted in accordance with the Declaration of Helsinki. The requirement for informed consent was waived by the committee because of the retrospective and observational nature of the study.

### 4.2. Data Collection and Microbiological Examinations

The data of each eligible patient, including their comorbidities, initial vital signs, laboratory results, microbiological profiles, management strategies, and outcomes, were collected from an anonymized electronic medical record system. The comorbidities were identified using specific disease codes (International Classification of Diseases, 10th Revision), and the initial ED vital signs and laboratory results were acquired within 6 h of the ED visit.

The isolated *K. pneumoniae* was cultured using the BACTEC 9240 automated detection blood culture system (Becton, Dickinson and Company Sparks, MD, USA) and identified using the VITEK 2 Compact system (bioMérieux, Inc., Marcy-l’Étoile, France). The further molecular characterization of *K. pneumoniae*, including polymerase chain reaction and deoxyribonucleic acid sequence analyses of drug-resistance genes, serotype and virulence genes, pulsed-field gel electrophoresis for clonality interpretation, and multilocus sequence typing for genetic relatedness and sequence variation measurement, were not performed in this study. Antibiotic susceptibility was determined using Gram-negative susceptibility cards on the Vitek system, and the minimal inhibitory concentration was interpreted according to the Clinical and Laboratory Standards Institute criteria [33].

### 4.3. Definitions

Chronic kidney disease was defined as a baseline estimated glomerular filtration rate of <60 mL/min/1.73 m^2^, which was obtained using the simplified Modification of Diet in Renal Disease equation [34]. Recent ICU admission was defined as ICU hospitalization in the previous six months, and prior antibiotic use was recognized if the patient received a specific antimicrobial agent in the previous three months before the infection. Infection sources were identified as follows: bloodstream, urinary tract (urinalysis revealing pyuria and bacteriuria), respiratory tract (new radiological infiltrate combined with clinically compatible symptoms), intra-abdominal infection, soft tissue infection, and others (minor infection sites). The SOFA score was calculated based on the worst parameters of organ dysfunction recorded within 6 h of the ED visit [35].

### 4.4. Outcome Measurement and Statistical Analyses

The primary outcome of this study was to investigate the risk factors for community-acquired CRKP infection in elderly patients, and the secondary outcome was to compare the risks of adverse outcomes between the CRKP and CSKP groups. We included ICU care, respiratory failure (i.e., mechanical ventilation), septic shock (i.e., requirement of vasopressors for hemodynamic support), and 90-day mortality as adverse outcomes. We also analyzed the risk factors for 90-day mortality in the community-acquired *K. pneumoniae* infection in elderly patients. Statistical Package for the Social Sciences (SPSS, Chicago, IL, USA), version 25.0, was used for all statistical analyses. For the continuous variables, the data were presented as the mean ± standard deviation with normal distribution or the median (interquartile range) with non-normal distribution and were compared using a two-sample t-test or Mann–Whitney U-test, respectively. The categorical variables were expressed as percentages and analyzed using the Chi-square or Fisher’s exact test. Univariate and multivariate logistic regression analyses were used to identify the risk factors for CRKP infection and 90-day mortality in community-acquired *K. pneumoniae* infection in elderly patients. Parameters with a *p* value < 0.1 in the univariate analysis were included in the forward, stepwise multivariate logistic regression analysis; age and sex were mandatory parameters in the final model, irrespective of the *p* value in the univariate analysis. A two-tailed *p* < 0.05 was considered statistically significant.

## Figures and Tables

**Table 1:** Clinical characteristics of elderly patients with *K. pneumoniae* infection enrolled from 1 January 2021, to 31 December 2021 at the E-Da Hospital, Kaohsiung, Taiwan (*n* = 528).

Variables	All (*n* = 528)	CRKP (*n* = 54)	CSKP (*n* = 474)	*p*-Value
Age, years, mean ± SD	77.1 ± 8.2	77.9 ± 8.6	77.0 ± 8.2	0.46
Male, *n* (%)	277 (52.5)	30 (55.6)	247 (52.1)	0.67
Comorbidities, *n* (%)				
Hypertension	370 (70.1)	40 (74.1)	330 (69.6)	0.54
Diabetes mellitus	287 (54.4)	27 (50.0)	260 (54.9)	0.57
Chronic kidney disease	221 (43.0)	30 (55.6)	191 (41.5)	0.06
Malignancy	136 (25.8)	16 (29.6)	120 (25.3)	0.51
Cerebrovascular accident	123 (23.3)	16 (29.6)	107 (22.6)	0.24
Recent ICU admission, *n* (%)	92 (17.4)	27 (50.0)	65 (13.7)	<0.01 *
Prior antibiotics use, *n* (%)				
Penicillin	76 (14.4)	13 (24.1)	63 (13.3)	0.04 *
Cephalosporin	68 (12.9)	10 (18.5)	58 (12.2)	0.20
Fluoroquinolone	62 (11.7)	10 (18.5)	52 (10.9)	0.12
Carbapenem	42 (7.9)	8 (14.8)	34 (7.2)	0.04 *
Infection source, *n* (%)				
Bloodstream	127 (24.0)	17 (31.5)	110 (23.2)	0.18
Urinary tract	210 (39.7)	19 (35.2)	191 (40.2)	0.56
Respiratory tract	97 (18.3)	11 (20.4)	86 (18.1)	0.71
Intra-abdomen	29 (5.5)	1 (1.9)	28 (5.9)	0.34
Soft tissue	57 (10.8)	5 (9.3)	52 (10.9)	0.82
Others	9 (1.7)	1 (1.9)	8 (1.7)	1.00
Laboratory results, median (IQR)				
Hemoglobin, g/dL	11.3 (9.4–13.3)	10.3 (8.8–12.4)	11.5 (9.6–13.3)	0.02 *
Leukocyte, ×10^9^/L	10.9 (7.3–14.8)	11.4 (7.4–15.7)	10.9 (7.3–14.8)	0.44
International normalized ratio	1.1 (1.0–1.2)	1.1 (1.0–1.2)	1.1 (1.0–1.2)	0.43
Bilirubin, mg/dL	0.9 (0.5–1.7)	0.5 (0.3–2.4)	0.9 (0.6–1.7)	0.08
C-reactive protein, mg/dL	69.3(21.6–146.8)	75.9 (26.3–133.7)	67.7 (21.2–148.6)	0.95
Creatinine, mg/dL	1.1 (0.8–1.7)	1.3 (0.8–2.1)	1.1 (0.8–1.7)	0.24
Combined antibiotics regimen, *n* (%)	276 (52.3)	30 (55.6)	246 (51.9)	0.38
SOFA score, mean ± SD	4 ± 3	5 ± 3	4 ± 3	0.03 *

* *p* < 0.05. SD, standard deviation; ICU, intensive care unit; IQR, interquartile range; SOFA, sepsis-related organ failure assessment; international normalized ratio, a blood clotting test/prothrombin assay; recent ICU admission, ICU hospitalization in the previous six months.

**Table 2:** Univariate and multivariate regression analysis of factors associated with carbapenem-resistant *Klebsiella pneumoniae* infection in elderly patients enrolled from 1 January 2021, to 31 December 2021 at E-Da Hospital, Kaohsiung, Taiwan (*n* = 528).

Variables	Univariate		Multivariate	
	OR (95% CI)	*p*-Value	OR (95% CI)	*p*-Value
Age (years)	1.01 (0.98–1.05)	0.47	1.01 (0.97–1.05)	0.55
Sex (male)	1.14 (0.65–1.72)	0.64	1.11 (0.57–1.85)	0.76
Chronic kidney disease Recent ICU admission	1.75 (0.99–3.09) 5.31 (3.48–7.43)	0.053 <0.01 *	1.48 (0.76–2.38) 7.48 (3.36–12.64)	0.25 <0.01 *
Prior penicillin use	2.07 (1.05–4.08)	0.04 *	2.06 (0.91–4.67)	0.09
Prior carbapenem use	2.26 (1.09–3.86)	0.04 *	4.01 (1.32–7.22)	0.02 *

* *p* < 0.05. OR, odds ratio; ICU, intensive care unit.

**Table 3:** Outcome analysis of elderly patients with *K. pneumoniae* infection enrolled from 1 January 2021, to 31 December 2021 at E-Da Hospital, Kaohsiung, Taiwan (*n* = 528).

Variables, *n* (%)	All (*n* = 528)	CRKP (*n* = 54)	CSKP (*n* = 474)	*p*-Value
ICU care	108 (20.5)	17 (31.5)	91 (19.2)	0.048 *
Respiratory failure	84 (15.9)	16 (29.6)	68 (14.3)	<0.01 *
Septic shock	121 (22.9)	19 (35.2)	102 (21.5)	0.03 *
90-day mortality	97 (18.4)	18 (33.3)	79 (16.6)	<0.01 *

* p < 0.05. ICU, intensive care unit.

**Table 4:** Univariate and multivariate regression analysis of factors associated with 90-day mortality in elderly patients with *K. pneumoniae* infection enrolled from 1 January 2021, to 31 December 2021 at E-Da Hospital, Kaohsiung, Taiwan (*n* = 528).

Variables	Univariate		Multivariate	
	OR (95% CI)	*p*-Value	OR (95% CI)	*p*-Value
Age (year)	1.01 (0.98–1.04)	0.54	1.01 (0.97–1.05)	0.75
Sex (male)	1.43 (0.92–1.74)	0.12	1.25 (0.61–2.58)	0.55
Chronic kidney disease	1.98 (1.26–2.80)	<0.01 *	1.07 (0.53–2.16)	0.86
Malignancy	1.75 (1.09–2.81)	0.02 *	1.98 (1.38–3.43)	<0.01 *
CRKP strain	2.51 (1.36–3.64)	<0.01 *	2.35 (1.92–3.06)	0.04 *
Recent ICU admission	5.16 (3.50–7.51)	<0.01 *	3.78 (0.66–5.81)	0.26
Bloodstream infection	1.75 (1.08–2.82)	0.02 *	1.69 (0.53–3.37)	0.37
Urinary tract infection	2.04 (1.25–3.33)	<0.01 *	1.31 (0.41–3.22)	0.65
Respiratory tract infection	2.00 (1.20–3.33)	<0.01 *	2.05 (0.60–3.96)	0.25
Hemoglobin	1.00 (0.98–1.03)	0.87		
INR	0.99 (0.92–1.07)	0.79		
C-reactive protein	1.004 (1.002–1.007)	<0.01 *	1.003 (0.99–1.007)	0.16
Creatinine	1.23 (1.10–1.39)	<0.01 *		
SOFA score	1.72 (1.55–1.90)	<0.01 *	1.64 (1.38–1.95)	<0.01 *

* p < 0.05. CRKP, carbapenem-resistant *Klebsiella pneumoniae*; ICU, intensive care unit; SOFA, sepsis-related organ failure assessment; INR, international normalized ratio.

## Data Availability

The datasets used and/or analyzed in the present study are available from the corresponding author upon reasonable request.
